# Endophytic *Bacillus subtilis* Strain E1R-J Is a Promising Biocontrol Agent for Wheat Powdery Mildew

**DOI:** 10.1155/2015/462645

**Published:** 2015-02-11

**Authors:** Xiaoning Gao, Yufei Gong, Yunxia Huo, Qingmei Han, Zhensheng Kang, Lili Huang

**Affiliations:** College of Plant Protection and State Key Laboratory of Crop Stress Biology for Arid Areas, Northwest A&F University, Yangling, Shaanxi 712100, China

## Abstract

In this study, the biocontrol efficacies of 14 endophytic bacterial strains were tested against *Blumeria graminis* f. sp. *tritici* (*Bgt*) in pot experiments under greenhouse conditions. *Bacillus subtilis* strain E1R-j significantly reduced disease index and exhibited the best control (90.97%). When different formulations of E1R-j were sprayed 24 h before *Bgt* inoculation, fermentation liquid without bacterial cell and crude protein suspension displayed the similar effects; and they reduced disease index more than bacterial cell suspension (10^9^ cfu mL^−1^) and fermentation liquid without protein. The control effects were not significantly different between 10^11^ and 10^9^ cfu mL^−1^ of bacterial cell suspension but were higher than 10^7^ cfu mL^−1^. Further observations showed that conidial germination and appressorial formation of *Bgt* were retarded by spraying E1R-j 24 h before *Bgt* inoculation. Compared with the water check, conidial germination and appressorial formation were decreased by 43.3% and 42.7%, respectively. In the treatment with E1R-j, the number of houstoria significantly reduced and the speed of mycelial extension was slowed down in the wheat leaves. Scanning electron microscopy observation revealed that E1R-j significantly suppressed the conidial germination and caused rupture and deformation of germ tubes. On the surface of wheat leaves, mycelia and conidiophores became shrinking.

## 1. Introduction

In cool and humid areas over the world, wheat powdery mildew, caused by* Blumeria graminis* (DC.) Speer f. sp.* tritici* Marchal (*Bgt*), is one of the most devastating diseases [1]. In China, severe epidemics were recorded on over 12 million ha which led to grain yield losses of approximately 1.4 and 0.7 million tons in 1990 and 1991, respectively [2, 3]. Since 2001, wheat powdery mildew has occurred annually on 6 to 8 million ha of wheat fields in China [4].

Growing resistant cultivars and/or applying fungicides are commonly used to control powdery mildew. Qualitative resistance, which is controlled by a single or few genes, is most widely utilized in wheat breeding programs. However, because qualitative resistance to powdery mildew is usually race-specific [1], its extensive development may lead to the rapid loss of effectiveness due to the coevolution of the pathogen's virulence [5]. Chemosynthetic fungicides play an important role in controlling wheat powdery mildew in China in the recent years. However, the development of pathogen strains resistant to the mostly used triadimefon fungicide has been reported and the resistant strain is now widespread [6, 7].

Biological control agents (BCAs), as an effective alternative control measure, are attracting more and more attention. Several microorganisms, for example,* Ampelomyces quisqualis*, have been found to be effective as biocontrol agents against various powdery mildew fungi [8–10]; and some are now available as commercial products in several countries [11, 12]. However, the studies and application of BCAs have mainly focused on the powdery mildew of vegetables and ornamentals in greenhouses [13–15], but little research has been conducted on control of wheat powdery mildew using BCAs. In the present study, we focused on the evaluation of the biocontrol effects of endophytic bacterial strains which were isolated from healthy wheat plants [16] against wheat powdery mildew under greenhouse conditions. We also studied the effects of bacterial strain E1R-j on infection of* Bgt* for understanding of the inhibition mechanisms.

## 2. Materials and Methods

### 2.1. Bacterial Strains, Wheat Cultivars, and* Bgt* Inoculation

Endophytic bacterial strains were isolated from healthy wheat plants [16] and stored at −80°C in 40% glycerol-containing nutrient broth-yeast extract (NBY). After activation from storage on Luria-Bertani agar, bacterial strains were inoculated into Luria-Bertani broth (100 mL in 250 mL Erlenmeyer flask) and incubated at 28°C for 48 h with constant shaking at 150 rpm. The bacterial culture was centrifuged at 12,000 rpm at 4°C for 20 min; the supernatant was used as the fermentation liquid without bacterial cells. The resulting residues were suspended in sterile distilled water (SDW) and the concentration was adjusted to 10^9^ colony forming units (cfu) mL^−1^ as bacterial cell suspension. Freshly prepared fermentation liquid without bacterial cells and bacterial cell suspension were used in each experiment.

Winter wheat (*Triticum aestivum* L.) cultivar Xiaoyan 6 was used in all experiments. About 10 wheat seeds were planted in each pot filled with a soil mixture (the soil and nursery substrate mixture of 3 : 1). Plants were grown in a growth chamber at 20°C, 70% relative humidity, with a 16 h photoperiod. Both seedlings at the two-leaf stage were used for experiments in the greenhouse.

Wheat plants maintained in a growth cabinet and heavily infected with* Bgt* were shaken one day before the harvest of spores to displace older spores and ensure that freshly formed conidia were available. Leaf segments bearing conidia were harvested and used for inoculation.

### 2.2. Greenhouse Experiments for Testing the Efficacies of Endophytic Bacterial Strains against* Bgt* on Wheat

In pot experiments, 14 endophytic bacterial strains were examined for their biocontrol efficacies against wheat powdery mildew. One leaf segment (with conidia) per pot was used to inoculate the leaves of the experimental plants (2-leave stage) by gently brushing the heavily infected leaf segment over the leaves to be inoculated, and then inoculated plants were incubated in a greenhouse. The fermentation liquid without bacterial cells and the bacterial cell suspension of experimental bacterial strain were sprayed separately on to the wheat leaves at 24 and 0 h before* Bgt* inoculation. Each treatment consisted of three pots.

Plant colonization by the fungus was quantified by measuring mildew colonies covering the surface of the leaves. Ten days after* Bgt* inoculation, disease severities were recorded according to the following scales: 0 = 0%; 1 ≤ 5%; 3 = 6%–15%; 5 = 16%–25%; 7 = 26%–50%; and 9 ≥ 50%.

The disease index and control efficacy were calculated as follows:

(1) Disease  index =  ∑number  of  diseased  stems  in  each  class1−1hhhhhHhhh×1−2number  of  the  severity  classeshhhhhhhhl×total  number  of  stems  investigated  hhhhhhhhhhhh×the  highest  severity  class−1×100;Control  efficacy  (%) =1−1disease  index  of  controlhhhhhh−1−2disease  index  of  treated  grouphhhlh×disease  index  of  control−1×100%.

### 2.3. Determination of the Biocontrol Effect of Different Formulations and Concentrations of Strain E1R-j on Wheat Powdery Mildew

Four formulations such as fermentation liquid without bacterial cells, bacterial cell suspension, crude protein suspension, and nonprotein fermentation liquid were used in this experiment. The preparation of fermentation liquid without bacterial cells and bacterial cell suspension was the same as described in Section 2.1. The crude protein suspension was produced by ammonium sulfate precipitation. Solid ammonium sulfate was gently added to the fermentation liquid to 70% relative saturation. The liquid was centrifuged at 12,000 rpm at 4°C for 20 min; the supernatant was collected and used as a formulation of nonprotein fermentation liquid. The precipitation was dissolved in 25 mM phosphate buffer (pH 7.0) and dialyzed extensively against distilled water to remove ammonium sulfate. The suspension was used as a formulation of crude protein suspension. Four formulations were especially sprayed on to the wheat leaves at 24 h before* Bgt* inoculation and 24 h after* Bgt* inoculation. Each treatment consisted of three pots (10 plants per pot) and the experiment was conducted three times. The cultivation of wheat plants, inoculation of* Bgt*, and the calculation of disease index and control efficacy were the same as described in Sections 2.1 and 2.2.

According to the described methods in Sections 2.1 and 2.2, the biocontrol efficacies of different concentration of bacterial cell suspension and fermentation liquid without bacterial cells of strain E1R-j were evaluated against* Bgt*. In this study, 10^7^, 10^9^, and 10^11^ cfu mL^−1^ of bacterial cell suspension were used. For the fermentation liquid without bacterial cells, the liquid was diluted to 0, 5- and 10-fold with sterile water.

### 2.4. Microscopic Examination of Effects of Strain E1R-j on Conidial Germination, Appressorial Formation, and Mycelial Extension of* Bgt*

Twenty-four hours after seedlings were sprayed with fermentation liquid without bacterial cells, the wheat plants were inoculated with* Bgt* conidia. The inoculated plants were kept in a condition-controlled greenhouse with the same temperature and light conditions as mentioned in Section 2.1. Wheat leaves were sampled at 8, 12, 24, 36, 48, 72, and 96 h after inoculation (hai). Leaves were treated using the method described by Li et al. [17]. The conidial germination, appressorial formation, haustorial development, and colony formation were observed using a light microscope (Leica DM LB2).

### 2.5. Observation of Inhibitory Effect of Strain E1R-j on* Bgt* Using Scanning Electronic Microscope

Wheat leaves were treated with fermentation liquid without bacterial cells and bacterial cell suspension. The other treatments were the same as described in Section 2.4. Wheat leaves were sampled at 12 h and 7 days after inoculation of* Bgt*. Leaves were treated using the method described by Kang [18]. The conidial germination and morphological variations of mycelia and conidiophores were observed under a JSM-6360 scanning electron microscope.

### 2.6. Data Analysis

Analysis of variance (ANOVA) was done for each set of disease data using the SPSS 16.0 (SPSS Inc., Shanghai, China). For the three independent repeated experiments, the data were tested firstly for significant differences. In fact, the repeated experiments did not result in significant differences and, therefore, the data were combined to test for differences among treatments. Among treatments, means were separated using the least significant difference (LSD) at *P* = 0.05.

## 3. Results

### 3.1. Effectiveness of Endophytic Bacterial Strains on* Bgt*

**Figure 1:**
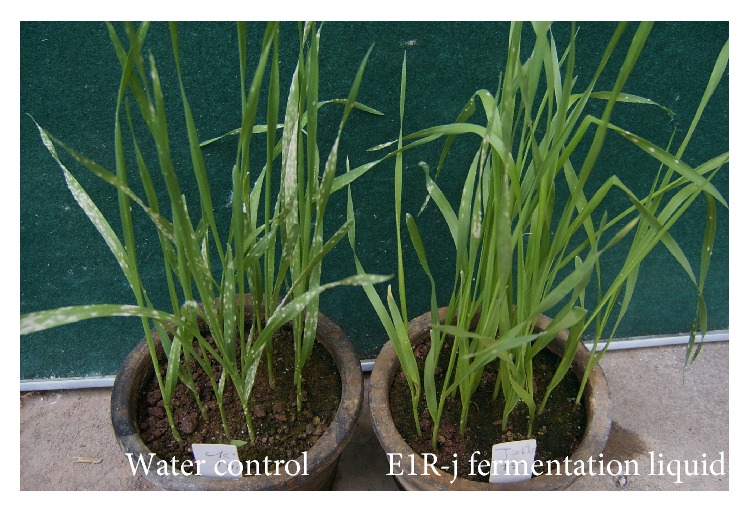
Disease severity of susceptible wheat cultivar Xiaoyan 6 seedlings 10 days after* Bgt* inoculation in the treatment of spraying strain E1R-j fermentation liquid 24 h before* Bgt* inoculation.

In the pot experiments, 14 endophytic bacterial strains, isolated from healthy wheat tissues, were screened for their ability to control powdery mildew of wheat seedlings in greenhouse assays. Through spraying the bacterial cell suspension and fermentation liquid without bacterial cells, four strains, E1R-j, E1R-h, ECL5, and Em7, showed higher effects (biocontrol efficacy > 55%) on wheat powdery mildew than other tested strains (Table 1). There were significant differences between the efficacies of bacterial cell suspension and fermentation liquid without bacterial cells of strains E1R-j, E1R-h, and ECL5. Among these treatments, the application of fermentation liquid of strain E1R-j resulted in the best control (Table 1). In addition, the time of spraying bacterial strains could affect the biocontrol efficacies. The control efficacies were higher when strains E1R-j, E1R-h, ECL5, and Em7 were applied 24 h before the fungal inoculation than at other times (Table 1). In conclusion, all treatments of strain E1R-j significantly reduced disease severity and showed the best control on* Bgt* among the test strains (Figure 1).

### 3.2. Effects of Strain E1R-j on* Bgt*

In order to further determine the effect of strain E1R-j on* Bgt*, the inhibition of bacterial cell suspension, fermentation liquid without bacterial cells, crude protein suspension, and nonprotein fermentation liquid was tested on wheat seedlings under greenhouse conditions. The results showed that all treatments reduced disease index compared to the water control (Table 2). When these suspensions were applied one day before the fungal inoculation, fermentation liquid without bacterial cells and crude protein suspension displayed similar effects on* Bgt* and the disease index decreased significantly more than the other treatments. The nonprotein fermentation liquid had the lowest control among all E1R-j treatments whenever the time of application was (either one day before or after the fungal inoculation).

Additional greenhouse tests have proven that the control efficacy was obviously influenced by the concentration of E1R-j cell suspension and dilution of fermentation liquid without bacterial cells (Table 3). The control effects were not significantly different between 10^11^ and 10^9^ cfu mL^−1^ of bacterial cell suspensions, but which was higher than 10^7^ cfu mL^−1^. For the fermentation liquid without bacterial cells, dilutions with 10-fold still significantly decreased the disease index. The maximum control efficacy was 63.84% treated with the 5-fold dilution of original formulation of fermentation liquid (Table 3).

### 3.3. Effects of Strain E1R-j on Conidial Germination, Appressorium Formation, and Mycelial Extension of* Bgt*

The inhibitory effects of the E1R-j fermentation liquid on the conidial germination and the appressorial formation of* Bgt* were recorded under light microscope. The microscopic observations of 8 h after inoculation showed that conidial germination of* Bgt* could be inhibited by spraying E1R-j 24 h prior to inoculation of* Bgt* (Table 4). In contrast with water control, the rate of conidial germination decreased by 43.3% in the treatment of strain E1R-j. The appressorial formation was observed 12 h after inoculation. The results indicated that E1R-j inhibited the appressorial formation of* Bgt* and the rate of appressorial formation was reduced to 43.3% (Table 4).

**Figure 2:**
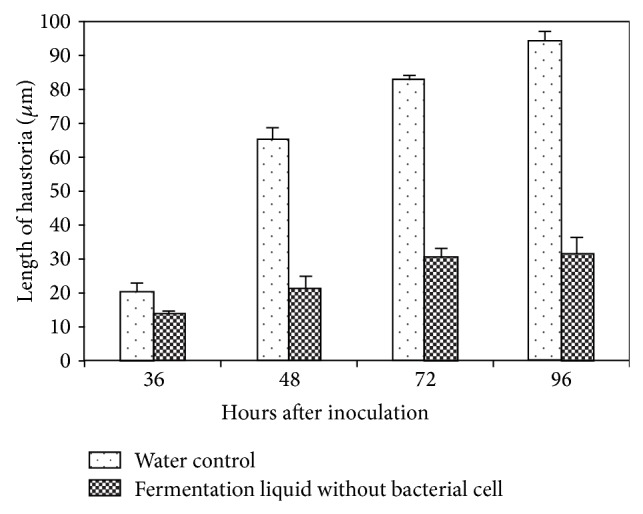
Effect of E1R-j fermentation liquid on the haustorial development of* Blumeria graminis *f. sp.* tritici*.

**Figure 3:**
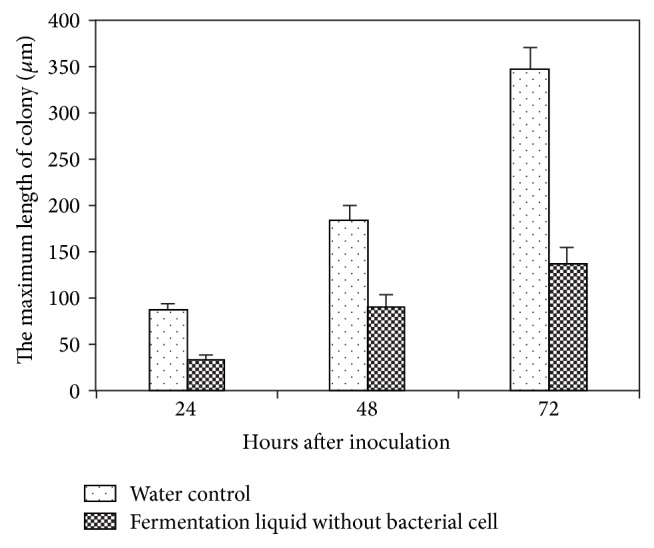
Effect of E1R-j fermentation liquid without bacterial cells on the colony length of* Blumeria graminis* f. sp.* tritici.*

The treatment of E1R-j fermentation liquid also suppressed the haustorial development and the mycelial extension. The microscopic observations showed that the average size of haustoria was up to 94.2 *μ*m in 96 hai in wheat leaves treated by water, but the number of haustoria was significantly reduced and the average size was 31.5 *μ*m in wheat leaves treated with E1R-j (Figure 2). The mycelial extension in the wheat leaves displayed the variation of quantity between the water control and E1R-j. The extension speed was slowed down by E1R-j (Figure 3).

### 3.4. Scanning Electronic Microscope Observation

**Figure 4:**
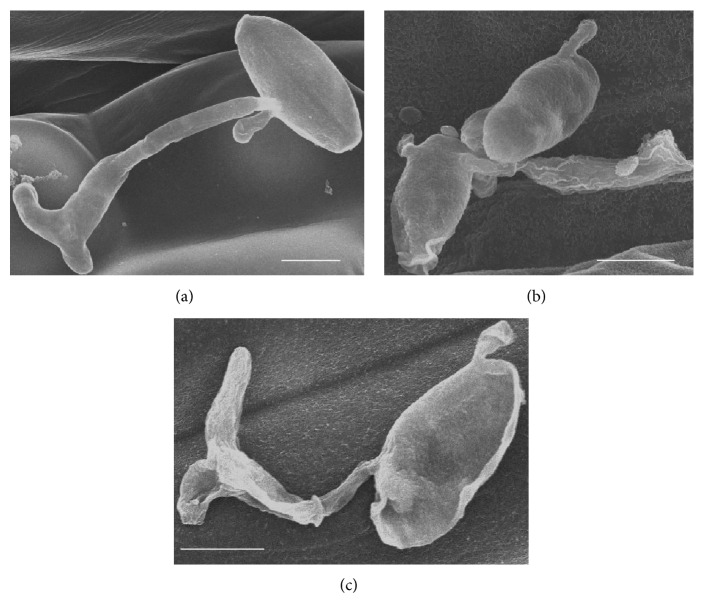
Conidial germination of* Blumeria graminis* f. sp.* tritici* (*Bgt*) on leaves of wheat cultivar “Xiaoyan 6” 12 h after inoculation of* Bgt* observed by scanning electron microscope. (a) Germination of conidia in the water control, also showing the germ tube formed; (b-c) conidia and germ tubes were destroyed by E1R-j cell suspension (b) and fermentation liquid (c). Bar = 10 *μ*m.

**Figure 5:**
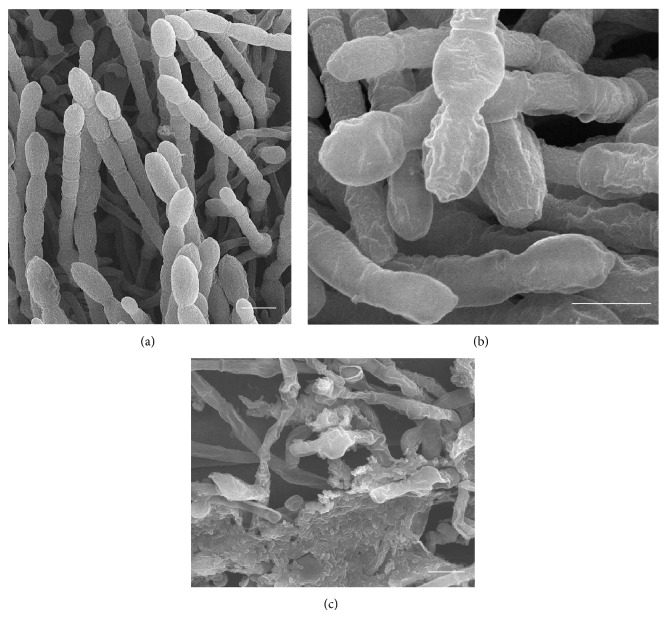
Morphological changes of* Blumeria graminis* f. sp.* tritici* (*Bgt*) treated with strain E1R-j on leaves of wheat cultivar “Xiaoyan 6” 7 days after inoculation of* Bgt* observed by scanning electron microscope. (a) The water control showing normal conidia morphology; (b) and (c) mycelia and conidiophore were destroyed and showed shrinking by fermentation liquid (b) and E1R-j cell suspension (c). Bar = 10 *μ*m.

Conidia germinated normally on the surface of wheat leaves sprayed with water (Figure 4(a)). In contrast, when treated with the bacterial cell suspension and fermentation liquid, conidia were ruptured or remained nongerminated and the germ tubes were damaged (Figures 4(b) and 4(c)). The morphology of* Bgt* on the surface of wheat leaves sprayed with the E1R-j bacterial cell suspension and fermentation liquid showed malformation. Mycelia and conidiophores displayed shrinking (Figures 5(b) and 5(c)). Some bacterial cells located on the mycelia of* Bgt* and the leaf surface (Figure 5(c)).

## 4. Discussion

Strain E1R-j was previously identified as* Bacillus subtilis* and had a broad spectrum against the important plant pathogens. Liu et al. [19] reported that E1R-j exhibited high antifungal activity to* Gaeumannomyces graminis* var.* tritici in vitro* and* in vivo*. Li et al. [20] determined that E1R-j had the inhibitory effect on wheat stripe rust in greenhouse and field trials. In the present study, E1R-j strongly inhibited* Bgt*. In the greenhouse experiments, the fermentation liquid without bacterial cells provided better control than the bacterial cell suspension. The results were consistent with the control of wheat stripe rust [20]. The postulated and demonstrated mechanisms of* Bacillus* spp. for controlling plant diseases included the production of metabolites with antimicrobial activity [21–23]. Therefore, we could conclude that the inhibitory effect is mainly due to the antifungal substances. Furthermore, we examined the efficacies of the crude protein suspension and nonprotein fermentation liquid. The crude protein suspension had a higher efficacy (89.04%) than the nonprotein fermentation liquid (16.17%); and there were no differences between crude protein suspension and fermentation liquid. The results suggest that E1R-j exerts its inhibitory activity through production of an antifungal protein. These results are in accordance with many reports that* Bacillus* species secrete proteins with antifungal activity [24, 25]. Future studies are needed to separate and purify the antifungal substance(s) of E1R-j and determine whether other inhibition mechanisms are existent.

The E1R-j fermentation liquid reduced disease index when sprayed before and after inoculation of* Bgt*. The results showed that E1R-j had dual actions (curative and protective effects) against* Bgt* and is consistent with microscopic observations. Microscopic observations revealed that the percentage of conidial germination and appressorial formation was significantly decreased in the presence of E1R-j fermentation liquid. E1R-j also perturbed conidial and appressorial morphogenesis. After penetration, the fungal growth can be affected by E1R-j produced substances. The development of haustoria and extension of mycelia were slowed down when E1R-j was sprayed.

In the previous study, E1R-j could colonize in the leaves and roots of wheat seedlings and effectively retarded infection and colonization of* Gaeumannomyces graminis* var.* tritici* in wheat root tissue [19]. In the present study, SEM observations showed that there were a large number of bacterial cells on the surface of sprayed leaves. However, it is unknown whether the colonization is a biocontrol mechanism of E1R-j against* Bgt*. Nevertheless, the present study laid the foundation for using endophytic bacteria to control wheat powdery mildew. In the future, E1R-j should be further investigated for control of wheat powdery mildew in the fields.

## 5. Conclusion

In this study, we firstly reported the biocontrol effect of endophytic bacterial strains on* Bgt*.* Bacillus subtilis* strain E1R-j showed a good control to wheat powdery mildew under greenhouse conditions. E1R-j not only inhibited conidial germination and appressorial formation but also inhibited the development of haustoria and extension of mycelia. Therefore, E1R-j can be used as a biological control agent to control powdery mildew on wheat.

## Figures and Tables

**Table 1:** Biocontrol efficacies of different endophytic bacterial strains on powdery mildew on seedlings of wheat in greenhouse.

Strains	Biocontrol efficacy of 10^9^ cfu mL^−1^bacterial cell suspension (%)^a^		Biocontrol efficacy of fermentation liquid without bacterial cell (%)	
	24 HBI^b^	0 HBI	24 HBI	0 HBI
E1R-j	64.21 ± 0.58	49.66 ± 0.14	90.97 ± 0.65	87.64 ± 1.10
E1R-h	65.29 ± 1.12	49.87 ± 0.58	85.21 ± 0.49	65.42 ± 0.83
ECL5	62.82 ± 0.52	55.21 ± 0.21	78.42 ± 1.13	69.32 ± 0.85
Em7	64.25 ± 0.38	55.47 ± 0.43	78.54 ± 0.67	56.85 ± 0.24
CW14	41.56 ± 2.16	^*^	36.48 ± 0.47	45.21 ± 0.68
EC6	35.47 ± 3.14	36.54 ± 1.21	48.64 ± 1.32	37.52 ± 0.78
ECS3	34.26 ± 0.23	8.377 ± 0.33	25.17 ± 0.74	^*^
ED21	30.71 ± 0.47	21.47 ± 0.94	^*^	15.17 ± 0.59
EDS14	21.79 ± 2.01	53.68 ± 0.75	39.68 ± 0.14	25.45 ± 0.47
B13	21.72 ± 0.95	41.74 ± 0.36	15.86 ± 0.69	25.14 ± 0.33
EM3	^*^	42.30 ± 0.42	42.13 ± 1.10	26.49 ± 0.45
EDR2	^*^	36.92 ± 0.98	32.57 ± 0.98	31.28 ± 0.56
EDF6	^*^	31.99 ± 1.42	16.84 ± 1.12	27.16 ± 1.75
CW8	^*^	10.95 ± 1.86	^*^	24.56 ± 1.47

SE, standard error of means.

^a^The biocontrol efficacy was calculated using the following formula: biocontrol efficacy (%) = (the disease index of water control − the index of treatment)/the disease index of water control × 100%. The disease indices were investigated 10 days after the inoculation of *Blumeria graminis* f. sp. *tritici*.

^b^HBI: hours before inoculation.

^*^The treatment did not have biocontrol effects.

**Table 2:** Control effect of *Bacillus subtilis *strain E1R-j on wheat powdery mildew in different treatments.

Treatments	24 hours before inoculation		24 hours after inoculation	
	Disease index^a^	Control efficacy (%)	Disease index	Control efficacy (%)
Water (control)	75.94 ± 1.21	—	75.94 ± 1.21	—
Crude protein suspension	8.37 ± 0.14	89.04 a^*^	29.34 ± 0.24	61.36 b
Fermentation liquid without bacterial cell	9.57 ± 0.24	87.40 a	9.76 ± 0.37	87.14 a
Bacterial cell suspension	44.65 ± 0.59	41.20 b	61.80 ± 0.76	18.62 c
Nonprotein fermentation liquid	63.66 ± 1.17	16.17 c	70.21 ± 1.56	7.55 d

SE, standard error of means.

^a^The severity of powdery mildew was recorded 10 days after *Blumeria graminis* f. sp. *tritici* inoculation. The disease index values were calculated using the formulas in Section 2.2.

^*^Means followed by the same letters within each treatment are not significantly different at the *P* < 0.05 level of confidence according to LSD's multiple range test.

**Table 3:** Biocontrol efficacies of different concentrations of bacterial cell suspension and fermentation liquid without bacterial cell of strain E1R-j on powdery mildew on seedlings of wheat tested in greenhouse.

Concentration of bacterial cell suspension (cfu mL^−1^)	10^11^	10^9^	10^7^	Water control
Disease index^a^	55.55	39.01	44.11	66.34
Control efficacy (%)	33.51 a^*^	41.20 a	16.27 b	—

Fold dilution of fermentation liquid without bacterial cell	0×	5×	10×	Water control

Disease index^a^	19.65	19.34	25.76	53.48
Control efficacy (%)	63.26 a^*^	63.84 a	51.83 b	

^a^The severity of powdery mildew was recorded 10 days after *Blumeria graminis* f. sp. *tritici* inoculation. The disease index values were calculated using the formulas in Section 2.2.

^*^Means followed by the same letters within each treatment are not significantly different at the *P* < 0.05 level of confidence according to LSD's multiple range test.

**Table 4:** The effect of fermentation liquid of strain E1R-j on conidial germination and appressorial formation of *Blumeria graminis* f. sp. *tritici *(*Bgt*).

Treatments	Conidial germination^a^			Appressorial formation^b^		
	Number of observed conidia	Number of germinated conidia	Percentage of germination (%)	Number of observed conidia	Number of appressoria	Percentage of formation (%)
Water control	150	141	93.3	150	129	86.0
Fermentation liquid without bacterial cell^c^	150	75	50.0	150	65	43.3

^a^The conidia germination was recorded 8 h after *Bgt* inoculation.

^b^The appressorial formation was recorded 24 h after *Bgt* inoculation.

^c^The fermentation liquid was sprayed on to the wheat leaves 24 h before inoculation *Bgt*.
